# MiR-203a-3p suppresses cell proliferation and metastasis through inhibiting LASP1 in nasopharyngeal carcinoma

**DOI:** 10.1186/s13046-017-0604-3

**Published:** 2017-10-05

**Authors:** Ning Jiang, Xuesong Jiang, Zhenzhang Chen, Xue Song, Lirong Wu, Dan Zong, Dan Song, Li Yin, Dejun Wang, Cheng Chen, Xiuhua Bian, Xia He

**Affiliations:** Department of Radiation Oncology, Jiangsu Cancer Hospital, Jiangsu Institute of Cancer Research, Nanjing Medical University Affiliated Cancer Hospital, 42 Baiziting Rd, Xuanwu District, Nanjing, 210000 Jiangsu Province People’s Republic of China

**Keywords:** Nasopharyngeal carcinoma, miR-203a-3p, LASP1, Proliferation, metastasis

## Abstract

**Background:**

miR-203a-3p was reported as a tumor suppressor and disregulated in many malignancies including nasopharyngeal carcinoma (NPC). However, its function in tumor growth and metastasis in NPC has rarely been reported.

**Methods:**

The expression level of miR-203a-3p in human NPC tissues and cell lines was detected via real-time PCR (RT-PCR). Cell proliferation, migration and invasion were assessed in vitro by MTT, colony formation and transwell assay, respectively. The function of miR-203a-3p in vivo was detected through NPC xenograft tumor growth and lung metastatic mice model. Dual-luciferase reporter assay was used to identify the direct target of miR-203a-3p.

**Results:**

The expression of miR-203a-3p was decreased in NPC tissues and cell lines in comparison with normal nasopharyngeal tissues and cell line. Ectopic expression of miR-203a-3p inhibited while inhibiting miR-203a-3p expression increased NPC cell proliferation, migration and invasion in vitro. MR-203a-3p overexpression suppressed xenograft tumor growth and lung metastasis in vivo. LASP1 was identified as a direct target of miR-203a-3p, which was confirmed by real-time PCR and western blotting assay. Ectopic expression of LASP1 partially reversed miR-203a-3p-mediated inhibition on proliferation, migration and invasion in NPC cells.

**Conclusion:**

Collectively, miR-203a-3p suppresses tumor growth and metastasis through targeting LASP1 in NPC. The newly identified miR-203a-3p/LASP1 pathway provides further insights into the initiation and progression of NPC, which may represent a novel therapeutic target for NPC.

## Background

Nasopharyngeal carcinoma (NPC) is an endemic malignancy in China [1]. There are over 60,000 new cases of NPC per year which caused 34,000 deaths every year in China [2]. Intensity-modulated radiation therapy (IMRT) and its combination with chemotherapy have greatly improved locoregional control of NPC, whereas tumor recurrence and distant metastasis remain as the main failure pattern and disease related death after treatment [3–5]. Thus, there is a great need to disclose molecular mechanisms that underlie the initiation and progression of NPC.

MicroRNAs (miRNAs) are a group of small non-coding RNAs which are dysregulated in many cancer types [6–11]. These newly identified regulators is involved in modulating cancer cell proliferation, differentiation, and migration through regulating genes’ expression by pairing with 3′-UTRs nucleotide sequences of their mRNAs [12–15]. They may function as both oncogenes and tumor suppressors. Up to now, a group of miRNAs have been reported participating in NPC progression and therapeutic response, such as miR-451 [16], miR-29c [17] and miR-19b-3p [18]. These findings indicate that miRNAs have important roles in nasopharyngeal tumorigenesis which worth further exploration.

MiR-203 has been reported down-regulated in NPC tissues through microarray analysis [19]. Recent studies found that miR-203 participated in NPC radioresistance and chemoresistance through negatively-regulate IL8/AKT pathway and ZEB2 [20, 21], respectively. However, its role in cancer cell growth and metastasis has rarely been characterized in NPC up to now. In this study, we found that the mature form of miR-203, miR-203a-3p, was downregulated in NPC tissues and could suppress cell proliferation and metastasis both in vitro and in vivo. Additionally, the LIM and SH3 domain protein (LASP1) was identified as a functional target of miR-203a-3p as previously reported in other malignancies such as esophageal squamous cell carcinoma, breast cancer etc. [22–28]. Thus, this study expands our understanding of the mechanisms underlying the development and progression of NPC, and may provide a novel therapeutic target for the treatment of NPC.

## Methods

### Cell lines and clinical specimens

Eight human NPC cell lines (CNE-1, CNE-2, C666–1, HNE-1, HONE-1, 5-8F, 6-10B and SUNE-1) were maintained in RPMI-1640 (Invitrogen, Grand Island, NY, USA) supplemented with 10% FBS (Gibco, Grand Island, NY, USA) [29]. A human immortalized nasopharyngeal epithelial cell line, NP-69, were cultured in keratinocyte/serum-free medium (Invitrogen) supplemented with bovine pituitary extract (BD Biosciences, San Diego, CA, USA). Sixteen freshly frozen NPC samples and seven normal nasopharyngeal epithelium samples were collected from Jiangsu Cancer Hospital (Nanjing, China). All samples were reviewed by pathologists to confirm the diagnosis. The research protocols were approved by the Institutional Ethical Review Board of Jiangsu Cancer Hospital, and informed consent was obtained from each patient. The expression profiles of miR-203a-3p were also investigated in NPC tissues obtained from Gene Expression Omnibus (GEO) (http://www.ncbi.nlm.nih.gov/geo).

### RNA extraction, reverse transcription and quantitative RT-PCR

Total RNA was extracted using TRIzol reagent (Invitrogen) as described previously [16]. Reverse transcribed using Bulge-Loop miRNA-specific RT primers (RiboBio, Guangzhou, China) for miR-203a-3p or random primers (Promega) for LASP1 with M-MLV reverse transcriptase (Promega, Madison, WI, USA). Quantitative RT-PCR reactions were performed on the ABI 7300 (Applied Bio-systems) using Platinum SYBR Green qPCR SuperMix-UDG reagents (Invitrogen). U6 or GAPDH were used as internal controls for miR-203a-3p and LASP1, respectively. The relative expression levels were calculated as previously described [16].

### Oligonucleotide and plasmid transfection

To explore the effect of miR-203a-3p on NPC cells, CNE-2 and SUNE-1 cells were transfected with miR-203a-3p mimic, miR-203a-3p inhibitor and their respective negative control(RuiboBio) using Lipofectamine 2000 reagent (Invitrogen). To determine that whether LASP1 is a direct target of miR-203a-3p, CNE-2 and SUNE-1 cells were co-transfected with miR-203a-3p mimic or miR-Ctrl (50 nM, RiboBio) and the pSin-EF2-puro-LASP1 (LASP1) or empty pSin-EF2-puro-Vector (Vector) (2 μg; Addgene, Cambridge, MA, USA). The cells were harvested for assays 48hs after transfection.

### Generation of stably transfected cell lines

The pre-miR-203a sequence was cloned into the lentiviral plasmid pSin-EF2-puromycin (Addgene, Cambridge, MA, USA); pSin-EF2-miR-203a or negative control pSin-EF2-vector was then co-transfected into 293FT cells with the psPAX2 packaging plasmid (Addgene) and the pMD2.G envelope plasmid (Addgene) using the calcium phosphate method. At 24 h after transfection, lentiviruses expressing miR-203a (Lenti-miR-203a) or negative control empty lenti-vector (Lenti-vector) were harvested and used to infect SUNE-1 cells, and stably transfected cells were selected using puromycin and validated by quantitative RT-PCR.

### MTT assay and colony formation assays

For the MTT assay, transfected CNE-2 or SUNE-1 cells were seeded in 96-well plates at a density of 1000 cells per well. The absorbance values were measured at 490 nm after 1, 2, 3, 4 and 5 days using an ELX800 spectrophotometric plate reader (Bio-Tek, Winooski, VT, USA). For the colony formation assay, cells were plated at a density of 500 cells per well in six-well plates after transfection, and cultured for 7 to 12 days. The colonies were then stained with 0.2% crystal violet with buffered formalin (Sigma, St. Louis, MO, USA). Colony numbers were manually counted using ImageJ software. Cell numbers >50 were considered as a colony.

### Wound healing assay and transwell migration and invasion assays

For the wound healing assay, transfected CNE-2 or SUNE-1 cells were seeded into 6-well plates and then subjected to serum starvation for 24 h in serum-free media. An artificial wound was created using a 200 μl pipette tip and images were taken at 0 and 24 h using an inverted microscope. Migration and invasion assays were performed in transwell chambers (Corning, Corning, NY, USA) coated with or without Matrigel (BD Biosciences) on the upper surface of the 8-μm pore size membrane. Briefly, transfected CNE-2 or SUNE-1 cells were harvested, suspended in serum-free medium and 5 × 10^4^ or 1 × 10^5^ cells were plated into the upper chamber for the migration or invasion assays, respectively. Media supplemented with 10% FBS was placed into the lower chamber. After 12 or 24 h incubation, the membranes were stained and counted using an inverted microscope.

### NPC xenograft tumor growth and lung metastasis mode in vivo

Male BALB/c nude mice of 4–6 weeks old were purchased from the Medical Experimental Animal Center of Guangdong Province (Guangzhou, China). For the xenograft tumor growth model, 1 × 10^6^ SUNE-1 cells stably overexpressing miR-203a or negative control were suspended in 200 μl PBS, and then subcutaneously injected into the dorsal flank of the nude mice. Tumor size was measured every 3 days, and tumor volumes were calculated as described previously [30]. Four weeks later, the mice were killed, and the tumors were dissected and weighted. For the metastasis assay, SUNE-1 cells stably overexpressing miR-203a or negative control were suspended in PBS, and 1 × 10^6^ cells (200 μl) were injected via the tail vein. Eight weeks later, the mice were killed, the lung tissues were fixed, paraffin embedded and 5 μm tissue sections were stained with hematoxylin and eosin (H&E). The number of macroscopic and microscopic metastatic nodules in the lungs was counted. All animal research protocols were approved by the Institutional Animal Care and Use Ethics Committee.

### Luciferase reporter assay

The LASP1 Wt and Mt. 3′-UTR were generated and cloned into the XhoI and NotI restriction sites of the psiCHECK-2 luciferase reporter plasmid (Promega). For the luciferase assay, CNE-2 or SUNE-1 cells were seeded into 6-well plates the day before transfection, and then co-transfected with the LASP1 Wt or Mt. 3′-UTR reporter plasmids (2 μg), and miR-203a-3p mimic (50 nM) or miR-Ctrl (50 nM) using Lipofectamine 2000 reagent (Invitrogen). Renilla and firefly luciferase activities were measured using the Dual-Luciferase Reporter Assay System (Promega).

### Western blot analysis

Cells were washed twice with PBS before being lysed on ice for 30 min with RIPA buffer containing protease inhibitor cocktail (Fdbio Science, Hangzhou, China). Protein concentrations were evaluated using the Pierce BCA Protein Assay Kit (Thermo Fisher Scientific, Waltham, MA, USA). Protein samples were separated by 10% SDS-PAGE gels and transferred onto Westran S membrane (Whatman Inc. Floham Park, NJ). The membranes were then incubated with mouse monoclonal anti-LASP1 antibody (1:2000; Chemicon, Temecula, CA, USA) and incubated with anti-mouse IgG secondary antibody (1: 5000; Epitomics, Burlingame, CA, USA). An anti-α-tubulin antibody (1: 1000; Sigma-Aldrich) was used as the loading control. Bound antibody was detected using the SuperSignal West Pico Chemoluminescence system (Pierce, Inc., Rockford, IL).

### Statistical analysis

Data are presented as mean ± S.D. All statistical analysis was performed using SPSS 20.0 software (IBM, Armonk, NY, USA). Two-tailed Student’s t-tests were used for comparisons between two groups. Comparison of means from multiple treatment groups was carried out using one-way ANOVA. A Bonferroni correction was introduced to correct for multiple comparisons. All *P* values were two-sided and *P* values less than 0.05 were considered significant.

## Results

### MiR-203a-3p is down-regulated in NPC cell lines and tissues

**Fig. 1:**
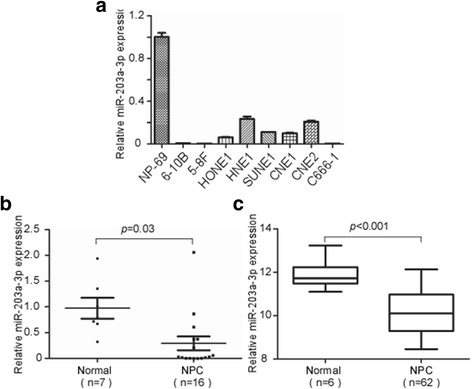
MiR-203a-3p is downregulated in NPC cell lines and clinical specimens. Relative expression of miR-203a-3p in NP69 and NPC cell lines by real-time PCR **a**. Relative expression of miR-203a-3p in normal nasopharyngeal epithelial (*n* = 7) and NPC tissues (*n* = 16) **b**. Each experiment was independently repeated for at least three times. Data are presented as mean ± S.D. MiR-203a-3p expression profile in 62 NPC and 6 normal nasopharyngeal epithelial samples using GEO database **c**. *P*-value was calculated using the Student’s t-test

MiR-203 was reported to be down-regulated in NPC tissues through high-throughput microarray assay [19].To confirm this result, we detected miR-203a-3p expression in both NPC cell lines and tissues. As shown by our results, miR-203a-3p expression was significantly downregulated in NPC cell lines when compared with the immortalized nasopharyngeal epithelial cell line NP-69 [31, 32] (Fig. 1a). Expression levels of miR-203a-3p were further investigated in NPC tissues, which was found to be significantly downregulated in NPC tissues as compared with normal nasopharyngeal epithelial tissues (Fig. 1b, *P* = 0.03). Moreover, data from the GEO database also confirmed that miR-203a-3p was significantly down-regulated in nasopharyngeal carcinoma (*n* = 62) in comparison with normal nasopharyngeal samples (n = 6) (GSE36682) (Fig. 1c, *P* < 0.001). These data suggest that miR-203a-3p was down-regulated in NPC and may function as a tumor suppressor.

### MiR-203a-3p suppresses NPC cell proliferation, invasion and migration in vitro

**Fig. 2:**
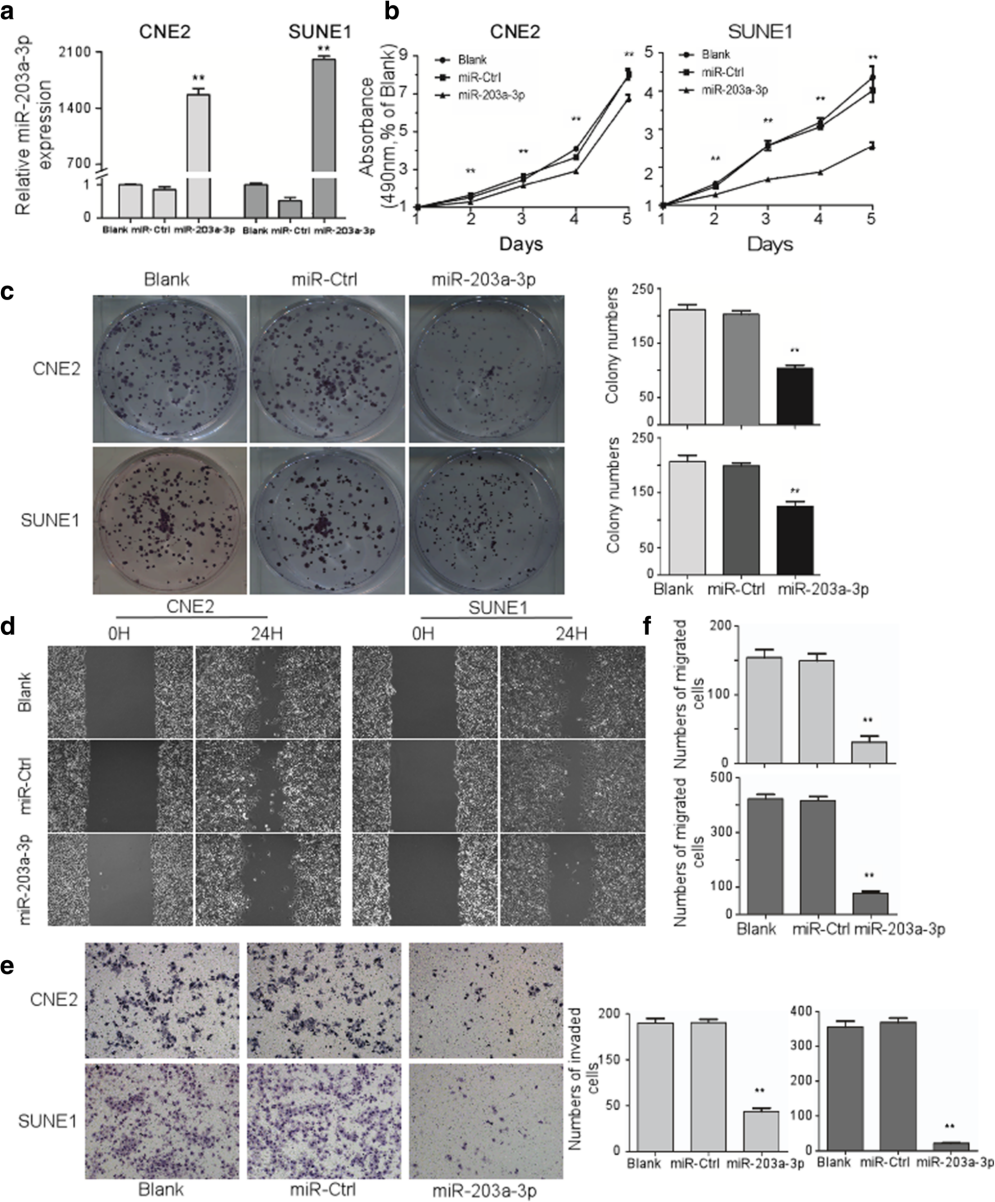
Ectopic expression of MiR-203a-3p suppresses NPC cell proliferation, invasion and migration in vitro*.* CNE2 and SUNE1 cells were transfected with miR-203a-3p mimic (50 nM), miR-Ctrl (50 nM), or the same volume of PBS (Blank). Expression of miR-203a-3p after transfection **a**. MTT assays were performed in CNE2 and SUNE1 cells on one to five days after transfection **b**. Colony formation was performed by crystal violet staining in CNE2 and SUNE1 cells **c**. Representative images for wound healing assay **d** and transwell invasion assay **e**. The cell counting results of transwell migration **f** and invasion assay **e**. * *P* < 0.05; ** *P* < 0.01 compared with miR-Ctrl or Blank groups. Each experiment was independently repeated at least three times. Data are presented as mean ± S.D. Statistical analysis was performed using one way ANOVA

**Fig. 3:**
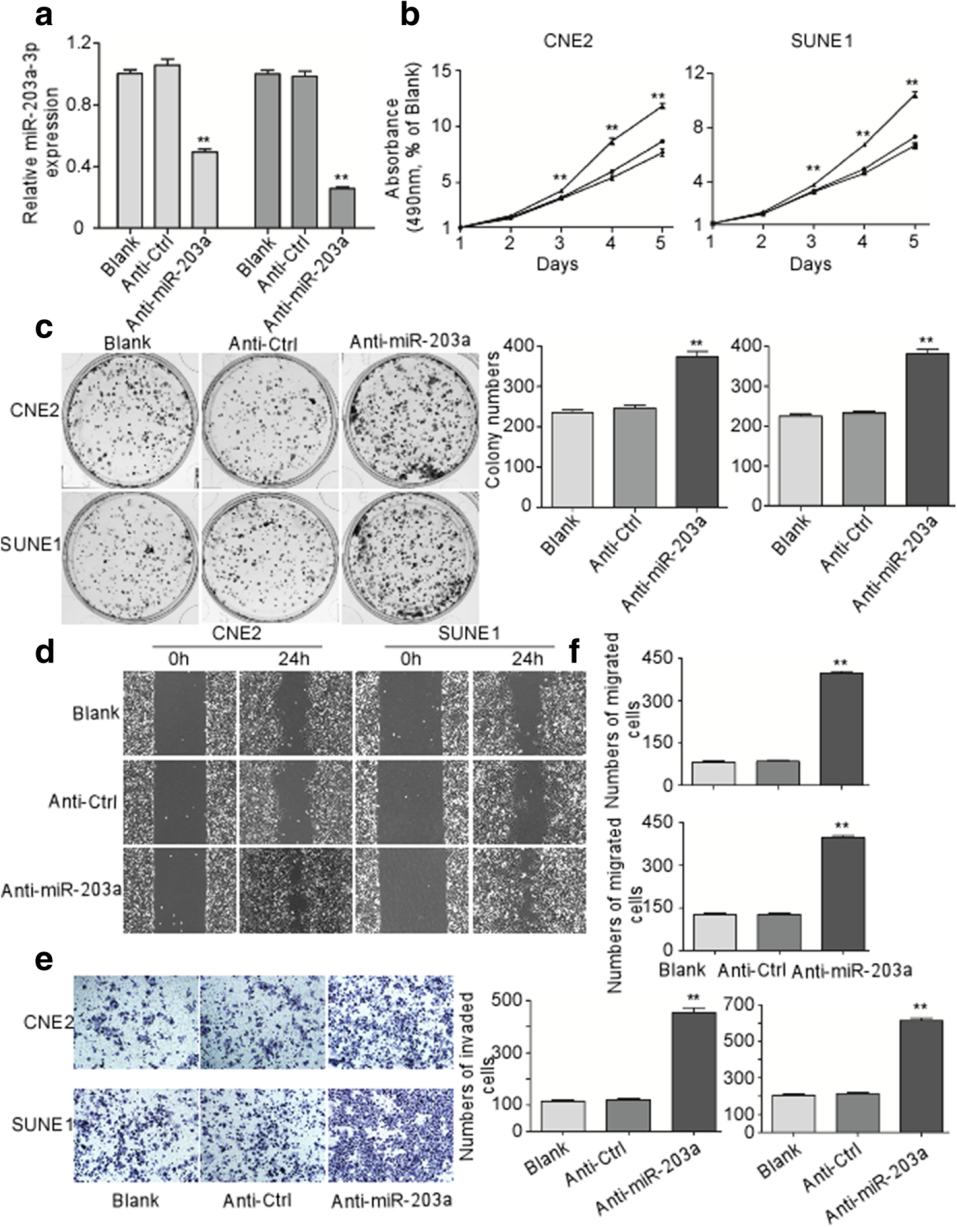
MiR-203a-3p inhibition increased NPC cell proliferation, invasion and migration in vitro*.* CNE2 and SUNE1 cells were transfected with miR-203a-3p inhibitor (Anti-miR-203a, 100 nM), negative control (Anti-Ctrl, 100 nM) or PBS (Blank). Expression of miR-203a-3p after transfection **a**. MTT assays were performed in CNE2 and SUNE1 cells on one to five days after transfection **b**. Colony formation was performed by crystal violet staining in CNE2 and SUNE1 cells **c**. Representative images for wound healing assay **d** and transwell invasion assays **e**. The cell counting results of transwell migration **e** and invasion assays **f**. ** *P* < 0.01 compared with Anti-Ctrl or Blank groups. The results are representative of at least three independent experiments. Statistical analysis was performed using one way ANOVA

The effects of miR-203a-3p on cell proliferation, migration and invasion were then evaluated in two representative NPC cell lines: CNE2 and SUNE1, through overexpressing (Fig. 2a) and inhibiting (Fig. 3a) miR-203a-3p expression. As shown in Fig. 2b and c, ectopically miR-203a-3p expression significantly inhibited proliferation and colony formation ability compared with the control groups in CNE2 and SUNE1 cells (*P* < 0.01). On the contrary, miR-203a-3p inhibition significantly increased cell proliferation (Fig. 3b *, P* < 0.01) and colony formation ability (Fig. 3c *P* < 0.01) in NPC in vitro. In addition, wound healing assay and transwell migration and invasion assays showed that up-regulation of miR-203a-3p significantly suppressed cell migration (Fig. 3d and f, *P* < 0.01) as well as invasion ability (Fig. 3e, *P* < 0.01) while inhibiting miR-203a-3p expression could enhance cell migration and invasion (Fig. 3d-f, *P* < 0.01) in the two NPC cell lines. Taken together, these results indicate that miR-203a-3p act as a tumor suppressor in NPC cell lines in vitro.

### MiR-203a-3p inhibits tumor growth and metastasis in NPC in vivo

**Fig. 4:**
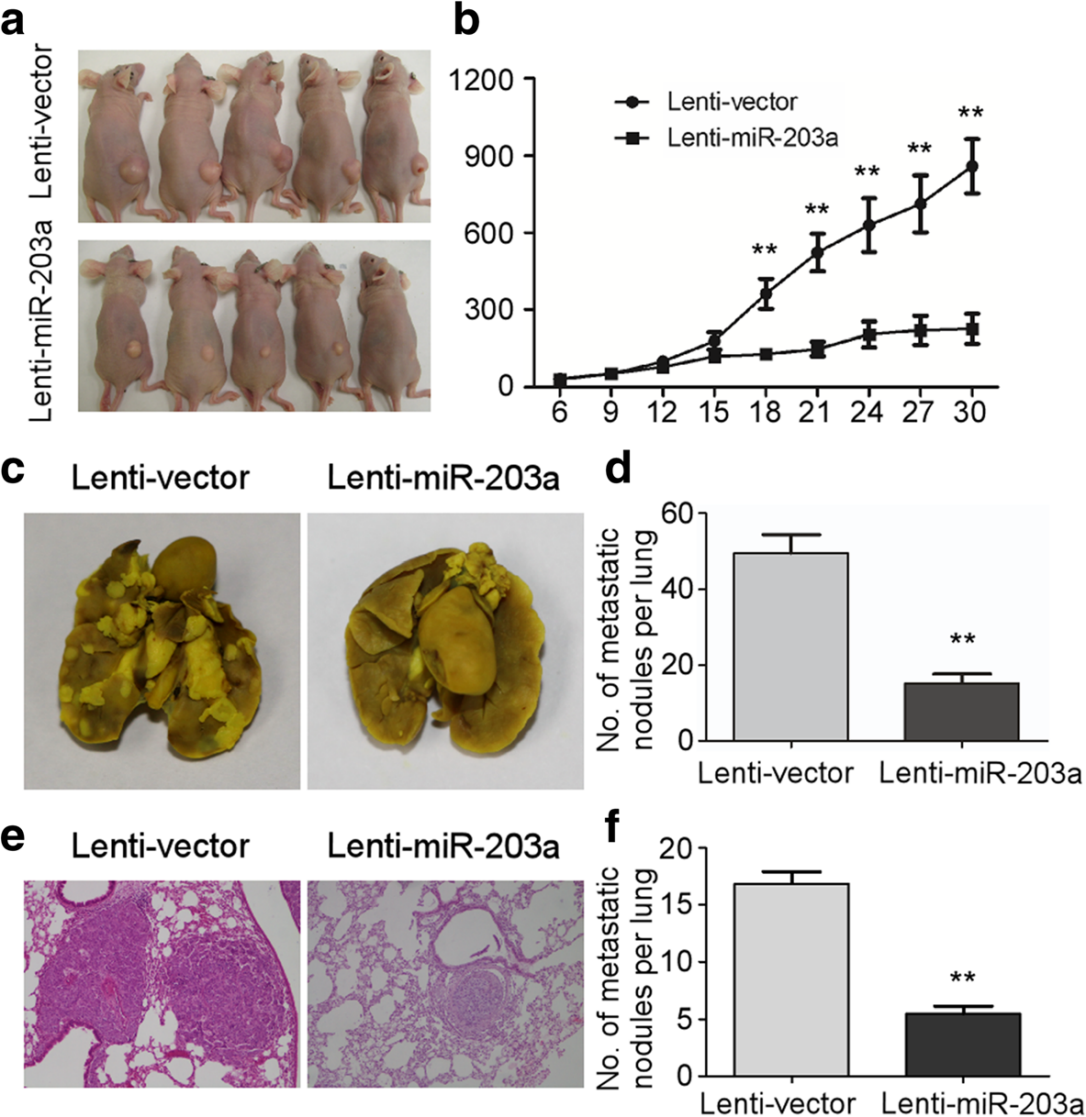
MiR-203a-3p inhibits tumor growth and metastasis in NPC in **vivo** ***.*** SUNE1 cells stably overexpressing miR-203a (Lenti-miR-203a) or negative control empty pSin-EF2-vector (Lenti-vector) were subcutaneously injected into right flank of each nude mouse (*n* = 6). A photograph of nude mice carrying tumors **a**. Volumes of all tumors were detected every 3 days **b**. SUNE-1 cells stably overexpressing miR-203a (Lenti-miR-203a) or negative control empty lenti-vector (Lenti-vector) were intravenously injected via the tail vein and the formation of lung metastases was assessed after 8 weeks. Representative images **c** and quantification **d** of macroscopic metastatic nodules on the lung surface. Representative images **e** and quantification **f** of microscopic metastatic nodules in lung tissue sections stained with hematoxylin and eosin (×100). Data are presented as mean ± S.D.; ** *P* < 0.01 compared with the Lenti-vector group, Student’s t-test

To further validate our data in vivo*,* we adopted the NPC xenograft model and lung metastatic model using SUNE-1 cell line. Firstly, we established the xenograft tumor model by subcutaneously injecting SUNE-1 cells stably overexpressing pre-miR-203a sequence (Lenti-miR-203a) or empty lenti-vector (Lenti-vector) into the dorsal flank of nude mice. Ectopic expression of miR-203a remarkably inhibited tumor growth 18 days after tumor formation (Fig. 4a and b; *P* < 0.01). For NPC lung metastatic model, cells were injected into nude mice via the tail vein. Eight weeks later, we found that lung metastatic nodules were significantly fewer in the miR-203a group than in the control group (Fig. 4c and d; *P* < 0.01). Microscopic observation of H&E staining slides revealed that metastatic nodules were significantly fewer and smaller in miR-203a group (Fig. 4e and f; *P* < 0.01). Taken together, these findings suggest that miR-203a suppresses tumor growth and metastasis in NPC in vivo.

### LASP1 is a direct target of miR-203a-3p in NPC cells

**Fig. 5:**
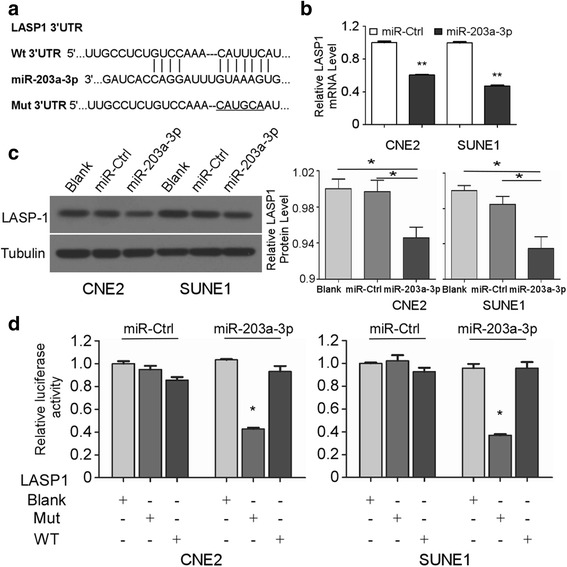
LASP1 is a direct target of miR-203a-3p in NPC cells. Wt or Mt. of the LASP1 mRNA 3′-UTR sequences targeted by miR-203a-3p **a**. Quantification of LASP1 mRNA levels by quantitative RT-PCR **b** and LASP1 protein expression by western blotting **c** after transfection with miR-203a-3p mimic or miR-Ctrl. Relative luciferase activity of CNE-2 and SUNE-1 cells after co-transfection with Wt or Mt. LASP1 3′-UTR reporter genes (2 μg) and miR-203a-3p mimic or miR-Ctrl (50 nM) **d**. Each experiment was independently repeated at least three times. Data are presented as mean ± S.D.; * *P* < 0.05; ** *P* < 0.01 compared with the miR-Ctrl group, Student’s t-test

To explore the molecular mechanism by which miR-203a-3p exerts its biological function, we identified LASP1 as a potential target for miR-203a-3p using two publicly available databases (Targetscan and miRanda, Fig. 5a). As shown in Fig. 5b and c, miR-203a-3p up-regulation inhibited LASP1 expression both on protein and mRNA levels. Furthermore, we generated constructed luciferase reporter vectors which contain the wild-type (Wt) or mutant (Mt) LASP1 3’-UTR sequences (Fig. 5a). When cells were transfected with the Wt LASP1 3’-UTR, co-transfection of miR-203a-3p inhibited luciferase activity significantly. In contrast, the inhibition was eliminated in cells co-transfected with the Mt. LASP1 3′-UTR (Fig. 5c and d). These findings suggest that LASP1 is a direct target of miR-203a-3p in NPC cells.

### LASP1 is involved in miR-203a-3p mediated tumor suppression

**Fig. 6:**
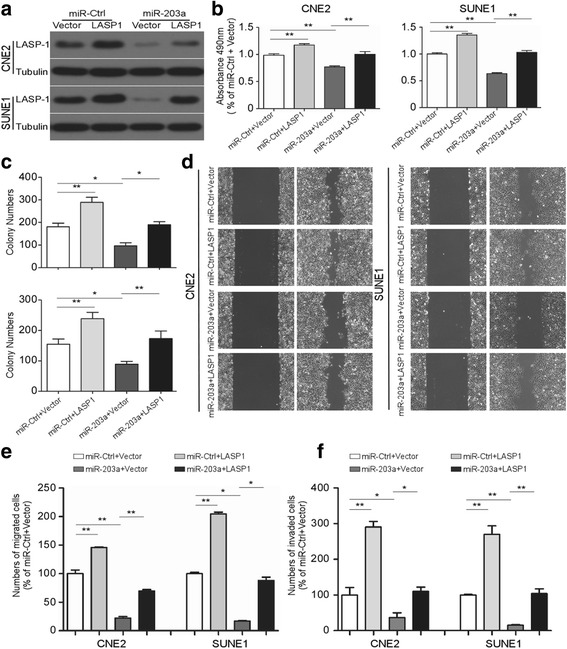
LASP1 is involved in miR-203a-3p mediated tumor suppression. CNE-2 and SUNE-1 cells were co-transfected with either miR-203a-3p mimic or negative control (miR-203a or miR-Ctrl) and either the pSin-EF2-puro-LASP1 (LASP1) plasmid or empty pSin-EF2-puro-Vector (Vector). **a** Western blotting analysis of LASP1 protein expression. **b**-**f** Overexpression of LASP1 abrogated the inhibition on cell proliferation, migration and invasion by miR-203a-3p. Representative results of the MTT assay (**b**), colony formation assay (**c**), wounding healing assay (**d**), transwell migration assay (**e**) and invasion assay (**f**). Data are presented as mean ± S.D.; * *P* < 0.05, ** *P* < 0.01, one way ANOVA. Each experiment was independently repeated at least three times

To further determine that LASP1 is one of the functional targets of miR-203a-3p, CNE2 and SUNE1 cells are co-transfected with either miR-203a-3p mimic or negative control (miR-203a or miR-Ctrl) and either the pSin-EF2-puro-LASP1 (LASP1) plasmid or empty pSin-EF2-puro-Vector (Vector) as control. LASP1 level was inhibited by ectopic miR-203a-3p expression, while LASP1 plasmid transfection restored its expression in NPC cells both in miR-203a and miR-ctrl groups (Fig. 6a). LASP1 up-regulation partially reversed the miR-203a-3p-mediated inhibition of cell proliferation (Fig. 6b, *P* < 0.01) and colony formation ability (Fig. 6c, *P* < 0.05). Moreover, up-regulation of LASP1 also abrogated the suppressive effects of miR-203a-3p on migration and invasion in NPC cells in vitro (Fig. 6d-f, *P* < 0.05). These results confirmed that miR-203a-3p mediated NPC tumor suppression through inhibiting LASP1.

## Discussion

MiR-203, which is now named as miR-203a, have been demonstrated to be an important tumor suppressor participating in the pathogenesis of esophageal [24, 33], prostate [23, 34], lung cancers [27, 35] including nasopharyngeal carcinoma [19–21]. A recent study found that miR-203 reduced radioresistance of NPC cells through inhibiting IL-8/AKT pathway [21]. In another investigation, miR-203 was reported modulating tumor stemness and chemotherapy resistance in nasopharyngeal carcinoma by interacting with ZEB2 [20]. However, miR-203 expression in nasopharyngeal carcinoma and its biological function on proliferation and metastasis have barely been investigated in NPC*.*

In the present study, we confirmed that the mature form of miR-203, miR-203a-3p, was down-regulated in both NPC cell lines and tissue samples. Ectopic expression of miR-203a-3p significantly suppressed cell proliferation, migration and invasion in vitro, and inhibited tumor growth and metastasis in NPC in vivo. Furthermore, LASP1 was identified as a direct target of miR-203a-3p in NPC. LASP1 over-expression partially abrogated the tumor suppression by miR-203a-3p in NPC cell lines. Taken together, our data suggest that miR-203a-3p/LASP1 pathway may play an important role in NPC initiation and progression.

MiRNAs are found dysregulated and lead to cancer initiation and progression in many types of cancers. Up to date, a group of miRNAs including miR-34c [36], miR-145 [37] and miR-93 [38] have been reported dysregulated and play important roles in NPC. A recent study screened for differentially expressed miRNAs in NPC tissues through high-throughput microarray assays and found that miR-203 may down-regulated in NPC tissues [19]. In the present study, we validated and confirmed this result in both NPC cell lines and tissues. We found that miR-203a-3p level was significantly lower in NPC cancer cells as compared to immortalized nasopharyngeal epithelial cell NP-69. Moreover, miR-203a-3p level was also decreased in NPC tissues in comparison with normal nasopharyngeal tissues. The dysregulation of miR-203a-3p indicated a potential tumor suppressor role in NPC.

We then explored the biological function of miR-203a-3p in NPC. Ectopic expression of miR-203a-3p significantly suppressed the proliferative, migratory and invasive capabilities of NPC cells in vitro. Furthermore, ectopic expression of miR-203a-3p inhibited NPC xenograft tumor growth and lung metastases in vivo. Up to now, this is the first study which confirmed the proliferation, migration and invasion suppressive effect of miR-203a-3p in NPC both in vitro and in vivo. Given our discovery that miR-203a-3p is frequently downregulated in NPC tissues, outlining a potential molecular target for NPC treatment.

LASP1, the LIM and SH3 protein-1, is a scaffold protein which mediates cell migration, proliferation and survival in various cancer entities [39–42]. In the present study, we identified LASP1 as a direct target of miR-203a-3p in nasopharyngeal carcinoma, in agreement with previous findings in head and neck cancer, breast cancer, esophageal carcinoma, prostate and lung cancers [22–28]. Ectopic expression of miR-203a-3p inhibited LASP1 level both on protein and mRNA level. In addition, ectopic expressing of LASP1 partially reversed the miR-203a-3p-mediated inhibition of proliferation, migration and invasion in NPC in vitro. These results indicate that LASP1 is a functional target of miR-203a-3p in regulating NPC cell proliferation and metastasis.

## Conclusions

In summary, our study demonstrated that miR-203a-3p is downregulated in NPC, and miR-203a-3p inhibited NPC cell proliferation and metastasis through targeting LASP1. MiR-203a-3p/LASP1 pathway may provide novel insight into the molecular mechanisms regulating progression of NPC, and may provide novel therapeutic targets for the treatment of NPC.

## Abbreviations

FBS: Fetal bovine serum
LASP1: The LIM and SH3 domain protein
MTT: 3-(4,5-Dimethylthiazol-2-yl)-2,5-diphenyltetrazolium bromide
NPC: Nasopharyngeal Carcinoma
PBS: Phosphate buffer saline
RT-PCR: Real-time polymerase chain reaction
